# Transcriptional and Distributional Profiling of Microglia in Retinal Angiomatous Proliferation

**DOI:** 10.3390/ijms23073443

**Published:** 2022-03-22

**Authors:** Anja Schlecht, Julian Wolf, Stefaniya Boneva, Gabriele Prinz, Barbara M. Braunger, Peter Wieghofer, Hansjürgen Agostini, Günther Schlunck, Clemens Lange

**Affiliations:** 1 Eye Center, Medical Center, Faculty of Medicine, University of Freiburg, 79106 Freiburg, Germany; julian.wolf@uniklinik-freiburg.de (J.W.); stefaniya.boneva@uniklinik-freiburg.de (S.B.); gabriele.prinz@uniklinik-freiburg.de (G.P.); hansjuergen.agostini@uniklinik-freiburg.de (H.A.); guenther.schlunck@uniklinik-freiburg.de (G.S.); 2 Institute of Anatomy and Cell Biology, Julius-Maximilians-University Wuerzburg, 97070 Wuerzburg, Germany; barbara.braunger@uni-wuerzburg.de; 3 Cellular Neuroanatomy, Institute of Theoretical Medicine, Medical Faculty, University of Augsburg, 86159 Augsburg, Germany; peter.wieghofer@medizin.uni-leipzig.de; 4 Ophtha-Lab, Department of Ophthalmology, St. Franziskus Hospital Muenster, 48145 Muenster, Germany

**Keywords:** AMD, Mactel 2, macular neovascularization, MNV type 3, retinal angiomatous proliferation, RAP, microglia, RNA sequencing, *Cx3cr1*, *CreERT2*

## Abstract

Macular neovascularization type 3, formerly known as retinal angiomatous proliferation (RAP), is a hallmark of age-related macular degeneration and is associated with an accumulation of myeloid cells, such as microglia (MG) and infiltrating blood-derived macrophages (MAC). However, the contribution of MG and MAC to the myeloid cell pool at RAP sites and their exact functions remain unknown. In this study, we combined a microglia-specific reporter mouse line with a mouse model for RAP to identify the contribution of MG and MAC to myeloid cell accumulation at RAP and determined the transcriptional profile of MG using RNA sequencing. We found that MG are the most abundant myeloid cell population around RAP, whereas MAC are rarely, if ever, associated with late stages of RAP. RNA sequencing of RAP-associated MG showed that differentially expressed genes mainly contribute to immune-associated processes, including chemotaxis and migration in early RAP and proliferative capacity in late RAP, which was confirmed by immunohistochemistry. Interestingly, MG upregulated only a few angiomodulatory factors, suggesting a rather low angiogenic potential. In summary, we showed that MG are the dominant myeloid cell population at RAP sites. Moreover, MG significantly altered their transcriptional profile during RAP formation, activating immune-associated processes and exhibiting enhanced proliferation, however, without showing substantial upregulation of angiomodulatory factors.

## 1. Introduction

Age-related macular degeneration (AMD) is the most common cause of irreversible vision loss in persons over age 65 in industrialized countries. It affects approximately 200 million people worldwide [1,2]. Common risk factors for AMD, such as age, cigarette smoking and a positive family history, have been identified [3], but a causal treatment has not yet been found. In advanced stages, 10–15% of patients develop neovascular AMD (nAMD), which is characterized by the formation of macular neovascularization (MNV), and rapidly leads to severe vision loss if left untreated. MNV type 3, also known as retinal angiomatous proliferation (RAP), was first described by Hartnett in 1996 and occurs in up to 15% of patients newly diagnosed with nAMD [4,5]. Similar to the neovascular lesions in patients with Macular telangiectasia type 2 (Mactel 2), MNV type 3 in nAMD originates from the retinal microvasculature and grows into the subretinal space, eventually leading to retino-choroidal anastomoses [4,6,7,8]. Experimental and clinical studies have shown that vascular endothelial growth factor (VEGF) plays a critical role in MNV formation and that inhibition of VEGF by anti-VEGF drugs is associated with beneficial effects on patient-relevant outcomes, such as visual acuity [9,10,11,12]. Similar to MNV types 1 and 2 (formerly known as occult and classical choroidal neovascularization), MNV type 3 can cause sight-threatening intraretinal edema and bleeding, resulting in irreversible loss of vision. In contrast to their choroidal counterparts, MNV type 3 lesions are less often associated with subretinal fibrosis and less responsive to anti-VEGF therapy [13,14,15,16]. The reasons for this clinical observation and the pathogenesis of MNV type 3 in general are still poorly understood. Age-related macular changes, ischemia and pro-angiogenic factors, such as VEGF, are currently thought to play a crucial role in the pathogenesis of RAP [17,18], but the cellular source of the pro-angiogenic mediators has not yet been identified.

In this context, myeloid cells are being discussed as significant contributors. However, opinions differ as to whether microglia or blood-derived macrophages dominate at RAP sites and whether one or the other has angiomodulatory properties and thus influences new vessel formation. Discrimination of resident microglia cells from infiltrating blood-derived macrophages during health, especially in the context of neovascular eye diseases, has been challenging for a lack of selective markers. The establishment of the tamoxifen-inducible *Cx3cr1^CreERT2^* reporter mouse line has greatly contributed to resolving some puzzles regarding the origin and discrimination of these two cell populations [19]. Recent studies by our group and other researchers showed that this reporter system not only provides a suitable model for tracing resident microglia and inflammatory macrophages in the retina during postnatal mouse development but also allows the discrimination of these cell populations in adult mice in both retina and brain [19,20,21]. In particular, this tool has been extremely useful in models of neovascular eye disease for examining the role of myeloid cells in choroidal and retinal neovascularization [20,21].

In this study, we crossed *Vldlr^−/−^* mice, developing RAP [22], to a microglia-specific reporter mouse line to determine if and to which proportion retinal MG and infiltrating MAC accumulate at the sites of RAP and to decipher the molecular signature of MG in this context. Our data suggest that retinal MG cells represent the major proportion of accumulating immune cells at sites of RAP, whereas blood-derived macrophages appear to play a minor role in terms of cell number. Furthermore, we provide evidence for strong MG activation in early RAP and MG proliferation at later stages, with little expression of angiogenic factors.

## 2. Results

### 2.1. Recombination Efficiency in Retinal Microglia and Blood of Vldlr^−/−^; MG^Tom^ Mice

**Figure 1:**
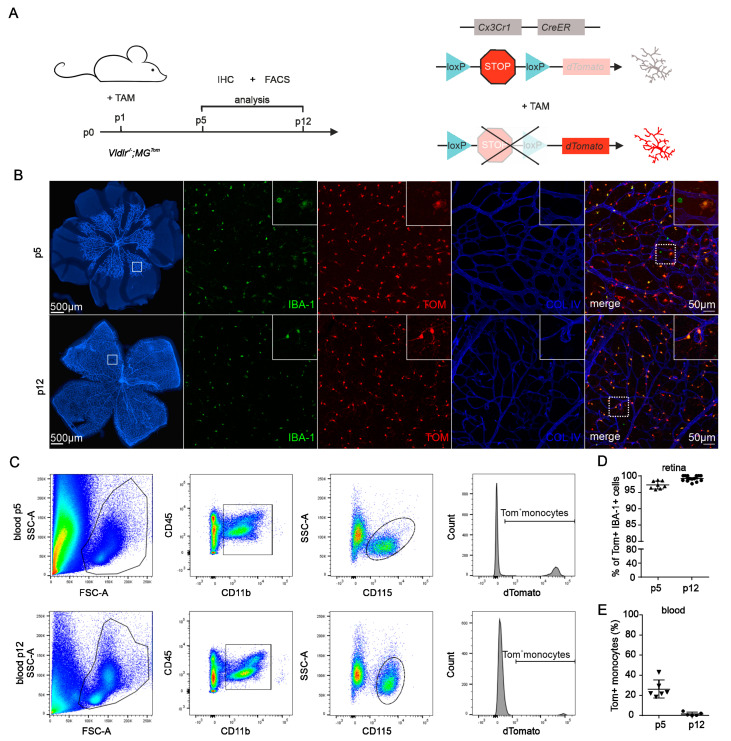
Recombination efficiency in retinal microglia and blood of *Vldlr^−/−^; MG^Tom^* mice. (**A**) Scheme displaying the experimental setup and timeline (left-hand side panel) for induction of reporter expression (right-hand side panel). Newborn *Vldlr^−/−^; MG^Tom^* mice were injected with tamoxifen (TAM) on postnatal day (p)1. Flow cytometry (FACS) and immunohistochemistry (IHC) were performed at p5 and p12. (**B**) Immunofluorescence of retinal vessels (Collagen IV (COLIV) staining, blue) and microglia (IBA1, green) at p5 and p12 following tamoxifen treatment at p1. TAM injection at p1 leads to efficient tdTomato (TOM, red) labeling of most microglia. In the top right corner of each image, a magnified image of the area within the dashed white box is displayed. (**C**) Flow cytometry analysis of the recombination efficiency in monocytes of *Vldlr^−/−^; MG^Tom^* mice at p5 and p12. Monocytes were gated as a CD45^hi^CD11b^+^CD115^+^Ssc^lo^ cell population from peripheral blood and analyzed for the proportion of TOM^+^ cells. FSC = forward scatter; SSC = sideward scatter. (**D**) Immunofluorescence-based quantification of TOM^+^ IBA1^+^ microglia of TAM-treated *Vldlr^−/−^; MG^Tom^* mice at p5 and p12. P5: *n* = 17, p12: *n* = 14 mice. (**E**) Flow cytometry-based quantification of TOM^+^ monocytes in the blood of *Vldlr^−/−^; MG^Tom^* mice. Data are presented as mean ± SD. p5: *n* = 6, p12 *n* = 5 mice.

To study the contribution of different myeloid cell subtypes to the development of neovascular eye diseases, we recently demonstrated that the *Cx3cr1^CreERT2/+^; Rosa26^tdTomato/+^* (short: *MG^Tom^*) mouse line is a suitable model to differentiate between microglia (MG) and peripheral blood-derived macrophages (MAC) in the retina of adults, as well as young mice [20,21,23]. To define the involvement of MG and MAC in the development of RAP, we crossed the *MG^Tom^* line with the *Vldlr^−/−^* mouse line (resulting in *Vldlr^−/−^; MG^Tom^*), which is regarded as a mouse model for RAP, with disease setting on postnatal day (p)15 [22]. First, we investigated the potential of this newly generated line in terms of recombination efficiency in MG and MAC. We assessed the recombination efficiency in retinal MG by immunohistochemistry and in MAC using FACS at p5 and p12 (Figure 1A). After tamoxifen injection at p1, *Vldlr^−/−^; MG^Tom^* mice revealed a high level of recombination events in retinal MG at p5 (mean ± SD = 97.2 ± 11.2%), reaching almost 100% at p12 (99.2 ± 0.8%), indicating a successful translocation of the Cre recombinase in retinal microglia (Figure 1B,D). Since *Cx3Cr1* is not only expressed in resident microglia but also in circulating monocytes of the peripheral blood, we next assessed TOM expression in CD45^hi^CD11b^+^CD115^+^SSc^lo^ peripheral monocytes at p5 and p12 in *Vldlr^−/−^; MG^Tom^* mice. While 26.3% (±8.9%) of peripheral monocytes still expressed TOM on day 5, the number of TOM-positive blood cells decreased substantially to only 1.5% (±1.8%) on day 12 (Figure 1C,E).

Thus, at the beginning of RAP formation at p15 [22], the Tomato fluorescent protein was expressed exclusively in retinal microglia, allowing a differentiation between retinal MG and infiltrating MAC during retinal angiomatous proliferation in the *Vldlr^−/−^* mouse.

### 2.2. Distributional Profiling of MG and MAC during Retinal Angiomatous Proliferation

**Figure 2:**
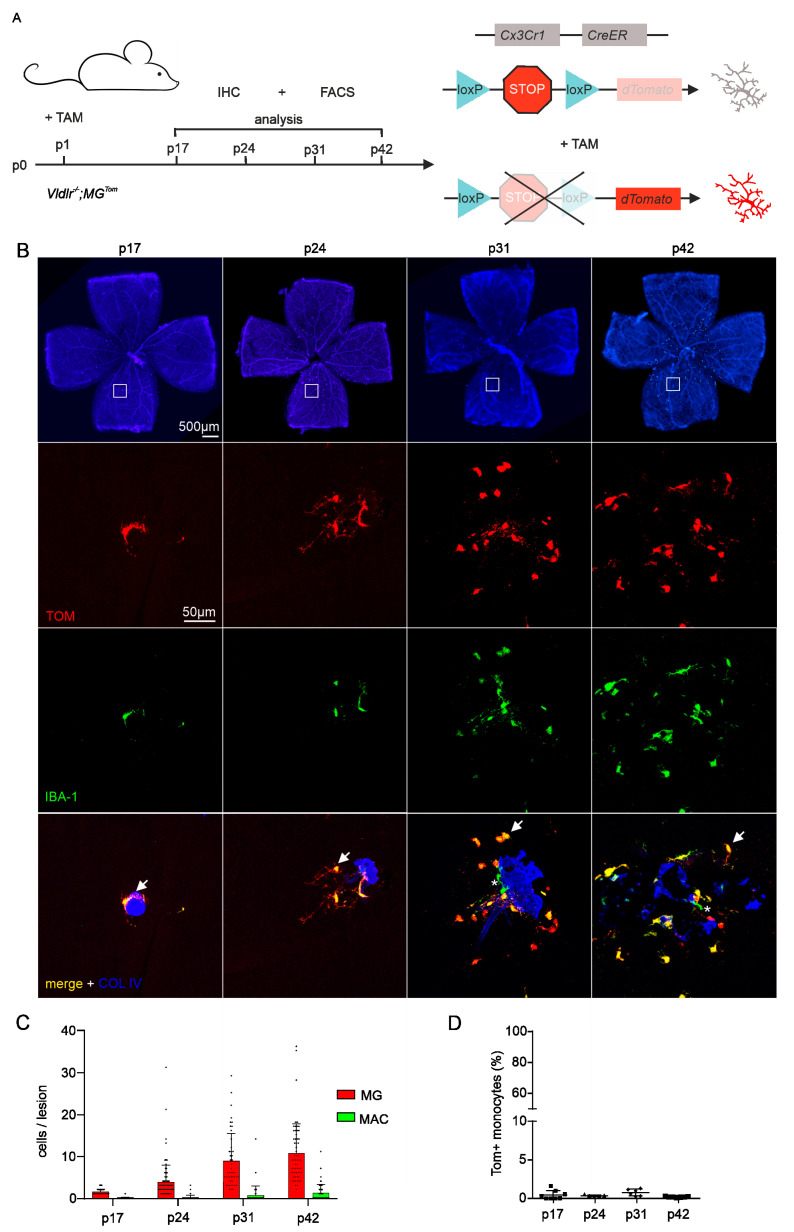
Distributional profiling of retinal microglia and blood-derived macrophages during retinal angiomatous proliferation (**A**) Scheme displaying the experimental setup and timeline (left-hand side panel) for induction of reporter expression (right-hand side panel). Newborn *Vldlr^−/−^; MG^Tom^* mice were injected with tamoxifen (TAM) on postnatal day (p)1. Flow cytometry (FACS) and immunohistochemistry (IHC) were performed at p17, p24, p31 and p42, respectively. (**B**) Upper panel: Retinal flat-mount stained for Collagen IV (COLIV, blue) demonstrating areas of retinal angiomatous proliferation (RAP), at p17, p24, p31 and p42, the areas within the white boxes are shown in higher magnification below: Representative confocal microscopy images of retinal angiomatous proliferation (COLIV, blue) and accumulating retinal microglia (defined as IBA1^+^TOM^+^) and infiltrating blood-derived macrophages (defined as IBA1^+^TOM^−^). Arrows point towards IBA1^+^TOM^+^ microglia and asterisks mark IBA1^+^TOM^−^ blood-derived macrophages. (**C**) Absolute numbers of microglia (MG) and respective blood-derived macrophages per RAP lesion quantified at p17, p24, p31 and p42. Red columns represent IBA1^+^TOM^+^ microglia, whereas green columns represent blood-derived IBA1^+^TOM^−^ macrophages. Data are presented as mean ± SD. Number of quantified RAP (N) = 75 (p17), 106 (p24), 65 (p31) and 60 (p42) lesions per group. For better visualization, one data point outlier (85 cells/per lesion, p31) was removed from the bar graph. (**D**) Flow cytometry-based quantification of TOM^+^ monocytes in the blood at p17, p24, p31 and p42 of *Vldlr^−/−^; MG^Tom^* mice. Data are presented as mean ± SD. N = 8 (p17), 5 (p24), 6 (p31) and 7 mice (p42).

Next, we investigated the numbers and relative distribution of MG and MAC accumulating at the sites of RAP lesions. For this purpose, we injected *Vldlr^−/−^; MG^Tom^* mice with tamoxifen at p1 and quantified the number of both cell populations in the course of RAP development at p17, p24, p31 and p42 (Figure 2A). Immunofluorescence of retinal flatmounts showed that the severity of RAP lesions increases with the age of mice (Figure 2B, lowest row). While the neovascular lesions tended to be solitary and rather small at p17 and p24, the RAP lesions appeared larger and showed pigmented areas at p31 and especially at p42. The age-dependent formation of RAP lesions was associated with an increase in the number of accumulating IBA1-positive myeloid cells (Figure 2B, middle rows). At p17, the onset of RAP formation, an average of 1.5 (±0.7) IBA1-positive myeloid cells was detected per lesion, with a slight increase to 3.9 (±4.3) myeloid cells per lesion at p24. At p31, the number of IBA1-positive myeloid cells accumulating at lesions increased to 10.3 (±12.2) cells per lesion and remained rather constant thereafter (p42: 11.8 ± 7.8; Figure 2C). Since *Vldlr^−/−^; MG^Tom^* mice revealed hardly any TOM-positive monocytes in the peripheral blood at all analyzed time points (p17: 0.5 ± 0.6%; p24: 0.4 ± 0.1%; p31: 0.8 ± 0.5%, p42: 0.2 ± 0.07%; Figure 2D), we next assessed the relative temporal distribution of Tomato-positive and IBA1-positive resident MG and Tomato-negative and IBA1-positive infiltrating MAC at sites of RAP. During early RAP formation at p17 and p24, only negligible numbers of infiltrating TOM^−^IBA^+^ MAC were found at the RAP lesions (p17: MAC = 0.03 ± 0.2 versus MG = 1.5 ± 0.7, *p* < 0.001; p24: MAC = 0.1 ± 0.5 versus MG = 3.8 ± 4.0, *p* < 0.001; Figure 2B,C). Late in the course of RAP formation at p31 or p42, we detected TOM^−^IBA^+^ MAC regularly near lesions, but still to a much lesser extent than resident microglia (p31: MAC = 0.5 ± 1.9 versus MG = 9.7 ± 11.2, *p* < 0.001; p42: MAC = 1.2 ± 2.0 versus MG = 10.6 ± 7.0, *p* < 0.001; Figure 2C). These data indicate that retinal microglia are the dominant myeloid cell population accumulating at RAP at all analyzed time points, while blood-derived macrophages quantitatively represent a subordinate cell population.

### 2.3. Transcriptional Profiling of Retinal Microglia during Early and Late RAP Formation

**Figure 3:**
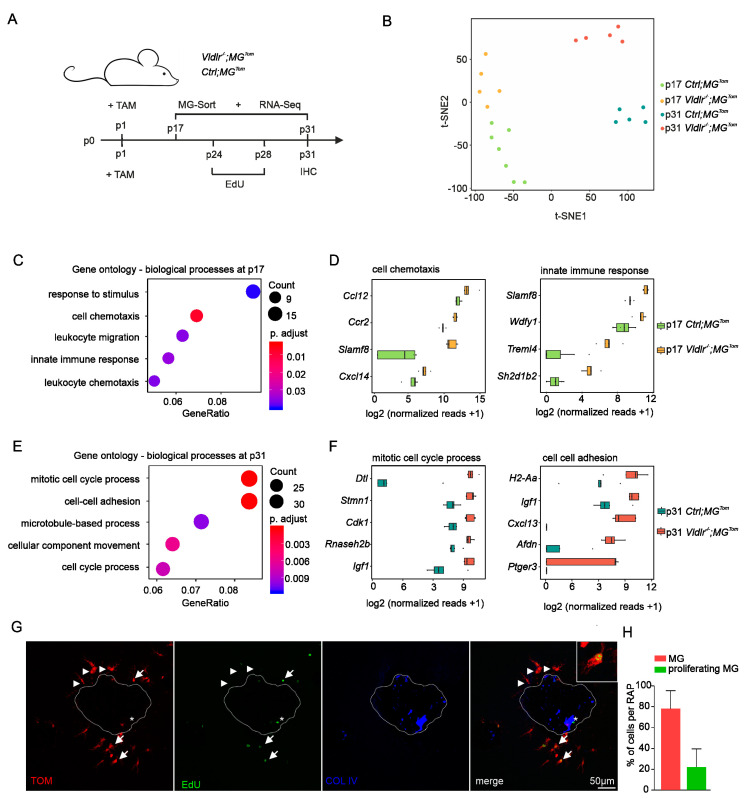
Transcriptional profiling of retinal microglia during early and late RAP formation. (**A**) Scheme displaying the experimental setup and timeline. Newborn *Vldlr^−/−^; MG^Tom^* and *Ctrl*; *MG^Tom^* mice were injected with tamoxifen (TAM) at postnatal day (p)1, CD45loCD11b+Ly6C-Ly6G-Tom+ microglia (MG) were isolated using FACS and analyzed by RNA sequencing (Seq) at p17 and p31. *Vldlr^−/−^; MG^Tom^* mice were injected with tamoxifen (TAM) at postnatal day (p)1, received daily EdU injections from p24 to p28 and finally were sacrificed for immunohistochemical analysis at p31. (**B**) T-distributed stochastic neighbor embedding of transcripts analyzed by RNA-seq in microglia from *Vldlr^−/−^; MG^Tom^* and *Ctrl*; *MG^Tom^* mice at postnatal day (p)17 and p31. (**C**–**F**) Gene ontology (GO) enrichment analyses of the 268 differentially expressed genes at p17 (**C**) and p31 (**E**). Dot plot depicting the top five enriched GO terms for biological processes in order of gene ratio. The size of the dots represents the number of genes in the differentially expressed gene list associated with the GO term, and the dots’ colors represent the *p*-adjusted values. Box plots illustrating the top enriched factors contributing to cell chemotaxis and innate immune response at p17 (**D**) and mitotic cell cycle process and cell–cell adhesion at p31 (**F**,**G**) Confocal microscopy images focusing on retinal microglia (TOM^+^, red) that have undergone mitosis (EdU^+^, green), accumulating around retinal angiomatous proliferation. Arrows point toward retinal microglia (TOM^+^, red) that have undergone proliferation (TOM^+^EdU^+^, yellow) and asterisks mark non-microglial cells, most likely endothelial cells, that have undergone mitosis (TOM^−^EdU^+^, green). Arrowheads point toward EdU-negative, nonproliferating microglia (TOM^+^EdU^−^, red). A higher magnification of a proliferating microglia cell (TOM^+^EdU^+^, yellow) is shown in the upper right corner. (**H**) Percentages of microglia with or without the EdU signal accumulating microglia around RAP at p31. Red columns represent non-proliferating and green columns represent proliferating microglia. Data represent mean ± SD. N = 79 RAP lesions.

Having established that retinal microglia are the predominant myeloid cell population accumulating at RAP, we next explored the transcriptional profile of MG during early and late RAP formation. To this end, we isolated CD45^low^CD11b^+^Ly6C^−^Ly6G^−^Tom^+^ retinal MG from tamoxifen-treated *Vldlr^−/−^; MG^Tom^* mice and respective *Vldlr*-competent *MG^Tom^* controls (*Ctrl; MG^Tom^*) at p17 and p31 and performed high-throughput next-generation RNA Seq for transcriptional analysis (Figure 3A). T-distributed stochastic neighbor embedding (t-SNE) revealed distinct clustering of all four groups, with no overlap between samples of different groups (Figure 3B). Applying strict thresholds (log2FC > 1.5 or < −1.5; adjusted *p*-value < 0.05) we found 268 differentially expressed genes (DEG) in the MG of *Vldlr^−/−^; MG^Tom^* compared to *Ctrl; MG^Tom^* mice during early RAP formation at p17, of which 125 were up- and 143 downregulated in the MG of *Vldlr^−/−^; MG^Tom^* mice. During late-stage RAP at p31, we identified 588 DEG, of which 354 were upregulated and 234 were downregulated in MG of *Vldlr^−/−^; MG^Tom^* mice. Gene ontology (GO) enrichment analysis revealed that DEGs during early RAP formation most significantly contribute to immune-associated processes, such as response to stimulus (GO:0035456, *p* = 1.5 × 10^−6^), cell chemotaxis (GO: 0060326, *p* = 6.3 × 10^−6^), leukocyte migration (GO: 0050900, *p* = 8.7 × 10^−5^) and chemotaxis (GO: 0030595, *p* = 1.0 × 10^−4^), as well as innate immune response (GO: 0045088, *p* = 7.0 × 10^−5^) (Figure 3C), indicating an immune cell activation and migration towards the lesions. The most prominent DEG contributing to cell chemotaxis and innate immune response are displayed in Figure 3D. *Ccl12* (log2FC = 1.9, p adj. = 2.9 × 10^−6^) and *Slamf8* (log2FC = 1.9, p adj. = 5.6 × 10^−16^) emerged as the top expressed genes in the respective GO terms. In the late course of RAP development, at p31, the transcriptional signature of MG showed a shift toward a profile associated with cell cycle and division processes, in particular mitotic cell cycle process (GO: 1903047, *p* = 1.5 × 10^−8^), cell-cell adhesion (GO: 0098609, *p* = 1.2 × 10^−7^), microtubule-based process (GO: 0007017, *p* = 4.8 × 10^−5^), cellular component movement (GO: 0051272, *p* = 7.2 × 10^−6^) and cell cycle process (GO: 0010564, *p* = 3.5 × 10^−5^) (Figure 3E). The most relevant factors for mitotic cell cycle process and cell–cell adhesion are shown in Figure 3F, including *Dtl, Stmn1* and *Cdk1*, as well as *H2-Aa*, *Igf1* and *Cxcl13*, respectively. These results are consistent with the large increase in immune cell accumulation around RAP lesions observed between p24 and p31 and suggest an enhanced proliferation of MG during late-stage RAP formation.

### 2.4. Proliferation of Retinal Microglia at Sites of RAP

To further assess the proliferation capacity of MG at sites of RAP, we administered the proliferation marker EdU daily from p24 to p28 and quantified the number of proliferating EdU^+^TOM^+^ microglia in the proximity of RAP lesions at p31. Using immunohistochemistry, we detected EdU^+^TOM^−^ cells in close association with the RAP lesions, which are most likely proliferating endothelial cells (Figure 3G, asterisk). Furthermore, we frequently detected EdU-positive cells, which also expressed Tomato and therefore represented proliferating retinal microglia (Figure 3G, arrows). Quantification of these cells showed that 22.8% (±17.4%) of the accumulating MG proliferated, which probably contributed to the substantial increase in cell numbers between p24 and p31 (Figure 2C and Figure 3H).

### 2.5. Angiogenic Potential of Retinal Microglia during RAP

**Figure 4:**
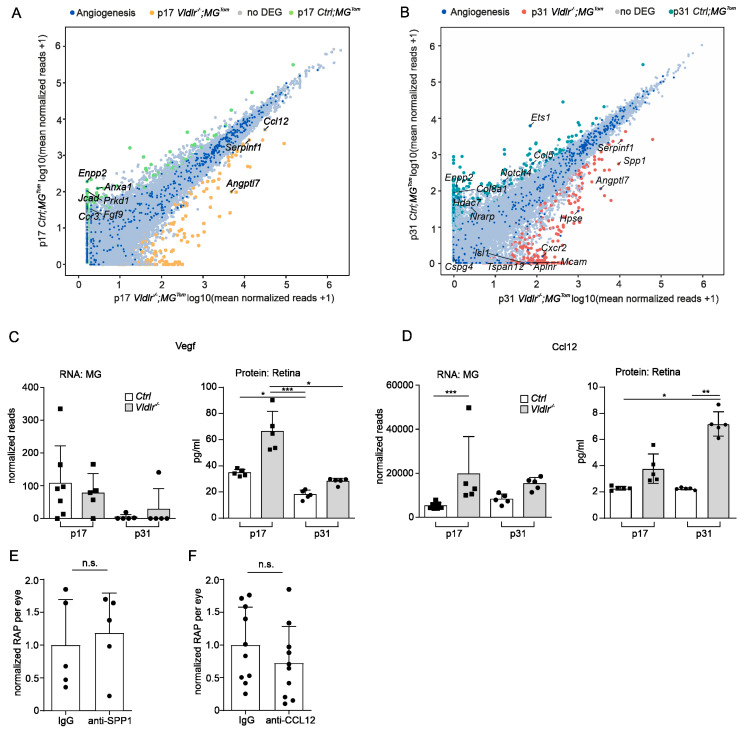
Angiogenic potential of retinal microglia during RAP. (**A**,**B**) Readplot using the log2 transformation of normalized reads visualizing differentially expressed genes between microglia from *Ctrl*; *MG^Tom^* mice (y-axis) and *Vldlr^−/−^; MG^Tom^* mice (x-axis) on postnatal day (p)17 (**A**) and p31 (**B**). Genes associated with angiogenesis are labeled blue. (**C**,**D**) Comparison of RNA Seq results of isolated microglia and protein analysis of retinal tissue lysates from *Vldlr^−/−^; MG^Tom^* and *Ctrl*; *MG^Tom^* mice at postnatal day (p)17 and p31 for *Vegf* (**C**) and *Ccl12* (**D**). mRNA: *n* = 5–7 samples per group, protein *n* = 5 samples per group. (**E**,**F**) Intravitreal injection of anti-SPP1 ((**E**), *n* = 5) or anti-CCL12 ((**F**), *n* = 10) in comparison to an IgG injection (*, *p* < 0.05; **, *p* < 0.01; ***, *p* < 0.001).

Considering that RAP formation is associated with increasing numbers of retinal MG at lesion sites, we next investigated whether MG also express factors that contribute to angiogenesis and may thus modulate disease progression. Based on the Gene Ontology term “angiogenesis” (GO: 0001525), we determined the genes expressed in retinal MG at RAP lesions at p17 and p31 (Figure 4A,B). Surprisingly, in the early phase of RAP development at p17, we found only 9 of 482 detected angiogenesis-associated genes to be differentially expressed; at p31, 16 factors out of 434 detected genes were defined as DEGs. In detail, at p17, retinal MG significantly upregulated only three angiogenesis-related genes. Among these, the chemokine (C-C motif) ligand 12 *(Ccl12)* was the most highly expressed (log2FC = 1.9, p adj. = 2.9 × 10^−6^), followed by *Serpinf1* (log2FC = 1.6, p adj. = 4.7 × 10^−13^) and angiopoietin-like 7 (*Angptl 7*, log2FC = 4.9, p adj. = 2.5 × 10^−7^) (Figure 4A,D). At p31, melanoma cell adhesion molecules (*Mcam,* log2FC = 8.7, p adj. = 0.04), C-X-C motif chemokine receptor 2 (*Cxcr2,* log2FC = 8.1, p adj. = 0.01), and heparanase (*Hpse,* log2FC = 5.3, p adj. = 0.02) also emerged as angiogenesis-related DEGs (Figure 4B). Furthermore, SPP1, which is a secreted angiogenic protein [24] modulating CNV formation [25,26] was significantly upregulated in MG at sites of RAPs at p31 compared to controls (*Spp1*, log2FC = 4.1, p adj. = 1.1 × 10^−5^). Since several studies describe increased VEGF-A expression in the retina of *Vldlr^−/−^* mice [27,28,29], we next investigated the expression of this well-established potent angiogenic factor in retinal microglia. Interestingly, *Vegf-a* was not defined as a DEG, neither in early- nor in late-phase RAP (p17: log2FC = −0.5, p adj. = 0.95; p31: log2FC = 2.8, p adj. = 0.63, Figure 4A–C). In contrast to RNA expression of *Vegf-a* in MG, which was barely detectable, we found robust expression of the VEGF-a protein, which was increased in the early phase of RAP at p17 in whole retinal lysates compared to controls (p17: Ctrl 34.7 pg/mL ± 2.6, *Vldlr^−/−^* 66.3 pg/mL ± 15.3; p31: Ctrl 18.1 pg/mL ± 3.5, *Vldlr^−/−^* 28.2 pg/mL ± 2.4). These data suggest a role for VEGF in RAP formation, which is produced by cells other than retinal MG. To explore whether *Ccl12* expression in retinal MG during RAP development is associated with a relevant CCL12 protein expression, we next quantified CCL12 protein expression in the retina. In accordance with the RNASeq data, we found increased CCL12 protein levels at p17 and p31 in *Vldlr^−/−^* mice compared to controls (p17: *Ctrl* 2.3 pg/mL ± 0.1, *Vldlr^−/−^* 3.7 pg/mL ± 1.1; p31: *Ctrl* 2.2 pg/mL ± 0.08, *Vldlr^−/−^* 7.2 pg/mL ± 0.9; *p* = 0.002 (p31)) (Figure 4D), suggesting that MG contribute to the increased CCL12 protein content during RAP formation. In summary, our data show that microglia express hardly any *Vegf-a* during RAP development but do express other angiogenic factors that might modulate disease progression. To test this hypothesis, we decided to inhibit the two most promising factors, namely SPP1 and Ccl12 by an intraocular injection of recombinant antibodies. Contralateral eyes were treated with an IgG control (Figure 4E,F). We found that inhibition of neither SPP1 nor Ccl12 resulted in significantly altered levels of RAP formation (SPP1: *Ctrl* = 1 ± 0.7 *Vldlr^−/−^* = 1.2 ± 0.6, *p* = 0.9, *n* = 5; Ccl12: *Ctrl* = 1 ± 0.6 *Vldlr^−/−^* = 0.7 ± 0.6, *p* = 0.9, *n* = 10).

## 3. Discussion

Retinal angiomatous proliferation (RAP), also known as macular neovascularization type 3, is present in up to 15% of patients with newly diagnosed neovascular AMD [4] and is associated with an accumulation of myeloid cells at the microscopic level, including resident microglia or blood-derived infiltrating macrophages [5,30]. Using MG-specific reporter mice and RNA sequencing of sorted retinal MG, this study revealed that retinal microglia proliferate during RAP formation and represent the dominant myeloid cell population at RAP compared with only a few infiltrating blood-derived macrophages. However, despite their close spatial association with proliferating endothelial cells, retinal microglia upregulate only a few angiomodulatory factors, suggesting no direct angiogenic effect of MG in RAP formation.

To discriminate MG from infiltrating macrophages, we combined the *Cx3cr1^CreERT2^* reporter mouse line with *Vldlr^−/−^* mice, a mouse model for retinal angiomatous proliferation. A thorough examination of this newly generated mouse line revealed that at postnatal day (p)12, before disease onset, almost all microglia cells expressed the reporter signal, whereas circulating monocytes in blood barely exhibited the fluorescent protein. This represents a crucial prerequisite for reliably distinguishing between resident microglial cells and infiltrating macrophages in order to study their function in the course of RAP formation. Quantification of the number of resident microglia and infiltrating macrophages at p17, p24, p31, and p42 revealed a substantial increase in the total number of myeloid cells over time. Our data also showed that, at all time points examined, microglial cells represent the dominant cell population at the RAP lesions, whereas infiltrating macrophages are found only rarely. This finding is consistent with recent studies showing that circulating monocytes from the blood rarely infiltrate the retina to accumulate at sites of neovascularization in both the oxygen-induced retinopathy and the laser-induced choroidal neovascularization mouse model [20,21].

Recent evidence suggests that myeloid cells modulate retinal neovascularization. As such, the depletion of myeloid cells in a mouse model of oxygen-induced retinopathy resulted in significantly reduced disease severity [31]. Similar results were recently published for the mouse model of retinal angiomatous proliferation [30]. Usui-Ouchi and colleagues elegantly demonstrated that genetic ablation of myeloid cells in *Vldlr^−/−^* mice resulted in a significantly lower number of RAP lesions compared with controls and that experimental activation of myeloid cells using LPS injection resulted in increased RAP formation [30]. Concomitant with reduced RAP formation following myeloid cell depletion, the authors reported that ablation of myeloid cells in this model rescued cone photoreceptor function [30]. However, the exact manner by which myeloid cells mediate these effects has not yet been definitively elucidated. To investigate this in detail, we isolated microglia at the onset and during progressive RAP formation and characterized the transcriptional profile of these cells using high-throughput RNA sequencing. Marked differences in the transcriptional profile of microglia upon RAP formation compared to controls were revealed, both in the early and late stages of the disease. GO cluster analysis showed that at the beginning of RAP development, factors associated with chemotaxis and migration, such as *Ccl12*, *Ccr2*, or *Cxcl14*, were highly upregulated, suggesting MG cell activation and migration toward the site of RAP. These data are consistent with an earlier study by our group investigating the transcriptional profile of microglia during retinal neovascularization in an oxygen-induced retinopathy mouse model [20]. According to our data, in the late course of RAP formation, proliferation processes dominate in microglia, concomitant with upregulation of factors such as *Cdk1* or *Igf1*, which have already been described as relevant factors for microglial proliferation [32,33]. This is consistent with our immunohistochemical analysis, showing an increase in proliferating retinal microglia in RAP lesions over time. Since our study indicates that retinal MG are the predominant cell population at sites of RAP, we next aimed to specifically investigate the expression of angiogenic factors in this myeloid cell subpopulation. Surprisingly, in this context, the expression profile of angiogenic factors in MG was hardly altered with RAP formation. At p17, only 9 of 482 detected angiogenesis-associated genes were differentially expressed, and at p31, 16 factors out of 434 detected genes. Moreover, the inhibition of two promising angiogenic factors (CCL12 and SPP1) by intraocular antibody injection did not alter RAP formation, indicating a rather low angiogenic potential of microglia in *Vldlr^−/−^* mice. Of note, while we found a significant increase in retinal VEGF-A protein in *Vldlr^−/−^* mice, RNA-seq data demonstrated that microglia hardly express any *Vegf-a* at p17, as well as at p31, and therefore do not directly contribute to VEGF-A-mediated RAP formation. This is consistent with the data of Usui-Ouchi et al. (2020), which revealed that myeloid cell-specific depletion of VEGF-A does not modulate RAP formation. The study by Usui-Ouchi and our study could be reconciled by yet unidentified MG-specific pro-angiogenic factors or by the hypothesis that infiltrating macrophages from the blood, which may also have been affected by pharmacological and genetic depletion [30] modulate RAP lesion formation. Additional studies analyzing the interaction of MG and infiltrating blood-derived macrophages, as well as the expression profile of MAC at RAP lesions, are needed to further investigate this hypothesis and to reliably determine the angiogenic potential of the different myeloid cell subpopulations and their potential as treatment targets during RAP formation. This may have future clinical implications for potential cell-specific immunomodulatory therapeutic approaches.

In summary, this study shows that retinal microglia are the dominant myeloid cells accumulating around RAP lesions. They strongly alter their transcriptional profile during RAP formation and express factors contributing to chemotaxis at disease onset and show increased proliferative potential as RAP formation progresses. However, contrary to the prevailing opinion, retinal MG upregulate few angiogenic factors during RAP formation, thus calling into question a major direct angiomodulatory effect of MG on RAP. Future studies using single-cell RNA-seq are warranted to investigate whether different subsets of retinal MG or infiltrating macrophages exhibit angiogenic properties or whether retinal MG accumulate at RAP to remove debris from surrounding dying neurons rather than directly promoting or inhibiting RAP.

## 4. Materials and Methods

### 4.1. Mice

All animal experiments conformed to EU Directive 2010/63/EU and were approved by the local authority (Regierungspräsidium Freiburg, Germany). *Vldlr^−/−^*, *Cx3cr1^CreERT2^* and *Rosa26-fl-stop-fl*-td*Tomato* (*Rosa26-tdTomato*) mice were bred under specific pathogen-free conditions on a C57BL/6J background. *Vldlr^−/−^* mice were crossed to *Vldlr^−/−^; Cx3cr1^CreERT2/CreERT2^*; *Rosa26^tdTomato/tdTomato^* mice to generate experimental *Vldlr^−/−^; Cx3cr1^CreERT2/+^; Rosa26^tdTomato/+^* mice. *Vldlr*-competent *Cx3cr1^CreERT2/+^; Rosa26^tdTomato/+^* mice served as controls for RNA sequencing (RNA-seq). For simplification, *Vldlr^−/−^; Cx3cr1^CreERT2/+^; Rosa26^tdTomato/+^* mice are hereafter referred to as *Vldlr^−/−^; MG^Tom^* mice and *Vldlr*-competent *Cx3cr1^CreERT2/+^; Rosa26^tdTomato/+^* mice as *Ctrl*; *MG^Tom^*. *Vldlr^−/−^* mice and respective C57BL/6J mice (controls) were used for protein analysis. All mice used in this study tested negative for the *Rd8* (Crb1) mutation.

### 4.2. Tamoxifen Treatment

CreER recombinase activity and subsequent tomato reporter expression were induced in experimental *Vldlr^−/−^; MG^Tom^* mice and in *MG^Tom^* mice and by a single subcutaneous injection of 5 µL (21 mg/mL) tamoxifen (T5648–1G, Sigma-Aldrich, Taufkirchen, Germany), dissolved in corn oil (C8267, Sigma-Aldrich) at p1, as previously described [20].

### 4.3. Fluorescence Microscopy

For fluorescence microscopy, the mice were perfused with PBS followed by 4% paraformaldehyde (PFA). After enucleation, eyes were fixed in 4% PFA for 1 h at 4 °C in the dark and retinal flatmounts were dissected prior to staining as previously described [34,35]. Primary antibodies against IBA1 (AB178846, Abcam, Cambridge, United Kingdom) and Collagen IV (COL4, AB769, Merck Millipore, Billerica, MA, USA) were added over two nights at a dilution of 1:500 at 4 °C in the dark. Secondary antibodies were added at a dilution of 1:500 (Alexa Fluor^®^ 647 and Alexa Flour^®^ 488, Life Technologies, Carlsbad, CA, USA) overnight at 4 °C in the dark. Retinal flatmounts were imaged using a Hamamatsu NanoZoomer S60 (Hamamatsu Photonics, Herrsching, Germany). To quantify microglia cell numbers, confocal images of RAP lesions were taken using a Leica SP8 confocal microscope with a 20× objective lens (Leica, Wetzlar, Germany). RAP lesions were detected when focusing below the outer plexiforme layer and were defined as neovascularizations originating from retinal vessels and extending into the normal avascular outer nuclear layer and reaching the retinal pigmented epithelium.

### 4.4. Identification of Microglia and Blood-Derived Macrophages

Confocal images of retinal areas (p5 and p12), as well as images of single RAP lesions (p17, p24, p31, p42), were taken as described above. IBA1^+^ cells were quantified at p5 and p12 per image (outer and inner plexiforme layer) and per RAP lesion at p17, p24, p31 and p42, respectively, using Fiji ImageJ [36]. Resident microglia cells were defined as IBA1-positive and tdTomato TOM-positive cells and blood-derived infiltrating MAC as IBA1-positive and TOM-negative cells. The proportion of TOM-positive and TOM-negative cells was determined relative to all IBA1^+^ cells.

### 4.5. In Vivo EdU Proliferation Assay

EdU (100 µg per day at a concentration of 1.25 mg/mL in PBS) was applied intraperitoneally on five consecutive days, from p24 to p28 in *Vldlr^−/−^; MG^Tom^* mice. Mice were sacrificed at p31 by intracardial perfusion. Fixation of the eyes and retinal flatmount preparation were performed as described above. The Click-iT™ EdU Alexa Fluor™ 647 Imaging Kit (C10340, Thermo Fisher Scientific, Waltham, MA, USA) was used to visualize EdU according to the manufacturer’s protocol, followed by Collagen IV immunohistochemical staining as described above.

### 4.6. Flow Cytometry

For sorting of retinal microglia cells, eye cups of PBS-perfused mice (p17, p31) were dissected to separate the retina from the underlying RPE/choroid. Retinal tissue was dissociated by resuspension. Dead cells were excluded by incubation in fixable viability dye 506 (65-0866-14, eBioscience, Thermo Fisher Scientific). Anti-CD16/CD32 Fc block (553142, BD Pharmingen, BD Biosciences, Franklin Lakes, NJ, USA) was performed at 4 °C for 20 min in the dark (1:200) to avoid unspecific binding. Afterwards, cells were stained with antibodies against CD45 (30-F11, 103133, BioLegend, San Diego, CA, USA), CD11b (M1/70, 17-0112-83, eBioscience, Thermo Fisher Scientific), Ly6C (AL-21, 45-5932-82, eBioscience, Thermo Fisher) and Ly6G (1A8, 551460, BD Pharmingen, BD Biosciences) at 4 °C for 20 min in the dark at a concentration of 1:200. Following staining, the cells were washed, and microglia cells were sorted using FACS Aria (BD Biosciences). To obtain an average of 4000 retinal microglia cells per sample for RNA sequencing analysis, the retinas of 1 to 2 mice were pooled. Retinal MG were sorted into, stored and shipped in RNA protect buffer (Quiagen, Germany) at 2–8 °C.

Peripheral blood samples were obtained by heart puncture prior to perfusion at p5 and p12. One hundred microliters of blood were lysed using RBC Lysis Buffer (00-4333-57, eBioscience, Thermo Fisher Scientific). Anti-CD16/CD32 Fc block (553142, BD Biosciences) was performed at 4 °C for 20 min in the dark (1:200) to avoid unspecific binding. Cells were stained with primary antibodies directed against CD45 (30-F11), CD11b (M1/70), CD115 (1:100, 25-1152-82, eBioscience, Thermo Fisher Scientific), Ly6C (AL-21) and Ly6G (1A8) at 4 °C for 20 min at a concentration of 1:200 in the dark. After washing, the cells were analyzed using a FACS Fortessa (BD Biosciences). Data were acquired with FACSDiva software (BD Pharmingen, BD Biosciences), and post-acquisition analysis was performed using FlowJo software (Tree Star, Inc., Ashland, OR, USA).

### 4.7. RNA Extraction

RNA extraction, library preparation and RNA-seq were performed at the Genomics Core Facility “KFB-Center of Excellence for Fluorescent Bioanalytics” (University of Regensburg, Germany; www.kfb-regensburg.de accessed on 25 January 2022). In brief, total RNA was extracted from isolated retinal microglia cells using the Rneasy Plus Micro Kit protocol (74004, QIAGEN, Hilden, Germany). After pelleting, the RNA protect buffer was removed and replaced by RLT Plus and the samples were homogenized by vortexing for 30 s. Genomic DNA contamination was eliminated using gDNA Eliminator spin columns. Next, ethanol was added, and the samples were applied to Rneasy MinElute spin columns, followed by several wash steps. Finally, total RNA was eluted in 12 μL of nuclease-free water. RNA purity and integrity were determined on an Agilent 2100 Bioanalyzer with the RNA 6000 Pico LabChip reagent set (5067-1513, Agilent, Palo Alto, CA, USA).

### 4.8. RNA Sequencing

In total, 22 microglia samples were analyzed using RNA Seq (p17: 5 RAP and 7 control samples; p31: 5 RAP and 5 control samples). The SMARTer Ultra Low Input RNA Kit for Sequencing v4 (Clontech Laboratories, Inc., Mountain View, CA, USA) was used to generate first-strand cDNA from 750 pg total RNA. Double-stranded cDNA was amplified by LD PCR (12 cycles) and purified via magnetic bead clean-up. Library preparation was carried out as described in the Illumina Nextera XT Sample Preparation Guide (Illumina, Inc., San Diego, CA, USA). In total, 150 pg of input cDNA was tagmented (tagged and fragmented) by the Nextera XT transposome. The products were purified and amplified via a limited-cycle PCR program to generate multiplexed sequencing libraries. For the PCR step 1:5 dilutions of index 1 (i7) and index 2 (i5) primers were used. The libraries were quantified using the KAPA SYBR FAST ABI Prism Library Quantification Kit (Kapa Biosystems, Inc., Woburn, MA, USA). Equimolar amounts of each library were pooled, and the pools were then used for cluster generation on the cBot with the TruSeq SR Cluster Kit v3 (GD-401-3001, Illumina, Cambridge, UK). The sequencing run was performed on a HiSeq 1000 instrument using the indexed 50-cycle single-read (SR) protocol and TruSeq SBS v3 Reagents according to the Illumina HiSeq 1000 System User Guide. Image analysis and base calling resulted in bcl files, which were converted into fastq files with the bcl2fastq v2.18 software.

### 4.9. Bioinformatics and Data Visualization

Sequencing data were uploaded to and analyzed on the Galaxy web platform (usegalaxy.eu), as previously described [37,38]. Quality control was performed with FastQC Galaxy Version 0.72 (http://www.bioinformatics.babraham.ac.uk/projects/fastqc/ last access on 10 April 2020). Reads were mapped to the mouse reference genome (Gencode [39], version M25) with RNA STAR [40] Galaxy Version 2.7.5b (default parameters) using the Gencode main annotation file (Gencode [39], version M25). Reads mapped to the mouse reference genome were counted using featureCounts Galaxy Version 1.6.4 [41] (default parameters) and the aforementioned annotation file.

The output of featureCounts was imported to RStudio (Version 1.2.1335, R Version 3.5.3). Gene symbols were determined based on ENSEMBL [42] release 101 (Mouse genes, download on 10 April 2020). Genes with 0 reads in all samples were removed from the analysis. Having confirmed distinct clustering of all 4 groups in t-distributed stochastic neighbor embedding (t-SNE) plots [43], differential gene expression was analyzed using the R package DESeq2 Version 1.22.2 (default parameters except using type = “iterate” in estimateSizeFactors) [44]. Transcripts with a log2fold change (log2FC) > 1.5 or <−1.5 and Benjamini-Hochberg adjusted *p*-value < 0.05 were considered differentially expressed genes (DEG). Gene enrichment analysis was performed using the R package clusterProfiler 3.10.1 [45]. Data visualization was performed using the ggplot2 package [46]. Genes involved in angiogenesis were selected based on the gene ontology terms “Angiogenesis” (GO: 0001525) (last accessed on 8 January 2020) [47].

### 4.10. Protein Analysis

Proteins were isolated from retinal or choroid tissue of p17 and p31 *Vldlr^−/−^* and control mice (*n* = 5 per group) using RIPA buffer (R0278, Sigma-Aldrich) containing protease (Complete Tablets Mini, 0463159001, Roche, Basel, Switzerland) and phosphatase inhibitors (Phosstop, 04906845001, Roche). The amount of isolated protein was measured using a colorimetric assay (Pierce™ BCA Protein Assay Kit, 23225, Thermo Fisher Scientific). To examine cytokine concentrations in retinal samples, we used a multiplex electrochemiluminescence panel (R-Plex, Meso-Scale Discovery, Rockville, MD, USA) according to the manufacturer’s instructions. This kit simultaneously measured the protein levels of CC-chemokine ligand (CCL12) and Vascular Endothelial Growth Factor A (VEGFA) in the same samples. These factors were chosen based on the RNA Seq results of this study and represented relevant or differentially expressed genes in RAP microglia compared to steady-state microglia. For statistical analysis, all values below the detection limit were assigned to half of the respective values of the detection limit.

### 4.11. Antibody Treatment

On postnatal day 15 (p15), before onset of RAP formation, one group of *Vldlr^−/−^* mice received an intravitreal injection of 50 ng anti-SPP1 (R&D Systems, AF808) solved in 1 µL PBS in one eye, whereas the contralateral eye was injected with the same amount of IgG control antibody (Nanofil Syringe 10 µL equipped with Nanofil 34G needle, World Precision Instruments, Sarasota, FL, USA). A second group of *Vldlr^−/−^* mice was injected with 1 ng anti-CCL12 (R&D Systems, AF428) into one eye with the same amount of IgG in the contralateral eye. The number of RAP lesions was quantified using COLIV staininf at p24, as described above.

### 4.12. Statistical Analysis

Statistical analysis was performed using GraphPad Prism (GraphPad Software, Version 6.0, La Jolla, CA, USA). Data were tested for normality applying the D’Agostino-Pearson Omnibus test and the Kruskal–Wallis or Mann–Whitney test was used for statistical analysis. Differences were considered significant when *p*-value < 0.05.

## Data Availability

Data are available from the corresponding author upon request.
